# Comparison of Infection-Induced SARS-CoV-2 Seroprevalence Across Large-Scale Residual Samples From Blood Donors, Commercial Laboratories, and Health Checkups in Japan, 2023

**DOI:** 10.1093/ofid/ofaf415

**Published:** 2025-07-17

**Authors:** Asuka Nishigaki, Ryo Kinoshita, Shoko Sakuraba, Jun Sugihara, Tadaki Suzuki, Motoi Suzuki, Daisuke Yoneoka

**Affiliations:** National Institute of Infectious Diseases, Japan Institute for Health Security, Tokyo, Japan; National Center for Global Health and Medicine, Japan Institute for Health Security, Tokyo, Japan; National Institute of Infectious Diseases, Japan Institute for Health Security, Tokyo, Japan; Ministry of Health, Labor and Welfare of Japan, Tokyo, Japan; Ministry of Health, Labor and Welfare of Japan, Tokyo, Japan; National Institute of Infectious Diseases, Japan Institute for Health Security, Tokyo, Japan; National Institute of Infectious Diseases, Japan Institute for Health Security, Tokyo, Japan; National Institute of Infectious Diseases, Japan Institute for Health Security, Tokyo, Japan

**Keywords:** COVID-19, SARS-CoV-2, seroepidemiology, seroprevalence, surveillance

## Abstract

We compared severe acute respiratory syndrome coronavirus 2 nucleocapsid antibody seroprevalence using 3 types of residual blood samples simultaneously collected in November–December 2023 from blood donors, commercial laboratories, and health checkups. Overall seroprevalence ranged from 53.5% to 58.8%, and notable variations were observed among the 30–39 and 50–59 age groups, highlighting the importance of multisource seroepidemiological surveillance.

The shift from notifiable to sentinel surveillance has posed challenges in accurately tracking the spread of severe acute respiratory syndrome coronavirus 2 (SARS-CoV-2). As many countries have scaled down extensive monitoring, seroepidemiological surveillance of SARS-CoV-2 nucleocapsid (N) antibodies has become essential for estimating the cumulative prevalence of SARS-CoV-2 infections, irrespective of symptom status [1, 2]. However, the impact of population characteristics on seroprevalence estimates remains poorly understood.

Random sampling is considered the gold standard for capturing seroprevalence in the general population [3, 4]; nonetheless, it is expensive and logistically challenging [5]. To address this, many countries have utilized residual serum samples from various sources, including blood donations [6–11], commercial laboratories [12–14], and health checkups [15]. Although residual samples are cost-effective and easy to collect, they may introduce a selection bias owing to the characteristics of the samples.

The general inclusion criteria are presented in Table 1. Blood donors are generally healthy individuals, selected through strict eligibility screening, including the absence of acute illness and certain chronic conditions [16]. Commercial laboratory samples are derived from individuals seeking health care, often for specific symptoms or medical concerns. Health checkup participants are typically employed individuals undergoing routine annual screenings mandated by the Industrial Safety and Health Act (with >80% of workers participating [17]) to identify chronic diseases or risk factors and are generally not acutely ill at the time of testing. These inherent differences in population characteristics may bias seroprevalence estimates, potentially leading to a misinterpretation of the actual burden on the general population. Previous studies have compared 2 specimen types: residual samples from commercial laboratories and blood donors, as well as random samples [18, 19]. Nevertheless, no studies have simultaneously examined all 3 sources.

**Table 1.** General Eligibility Criteria for Inclusion for Each Specimen Source in Japan

Specimen Source	Age Range, y	Target Population	Inclusion Criteria
Blood donor	16–69	Healthy population	Eligible to donate blood based on blood center guidelines (eg, minimum hemoglobin level, no recent infections or surgeries, no chronic conditions, no high-risk behaviors) [7]
Commercial laboratory^a^	≥0	Hospital attendants	Referred for laboratory tests by a health care provider May include individuals with underlying health conditions No specific exclusion based on health status
Health checkup^a^	≥15	Working population	Enrolled in a health checkup program Employed worker No specific exclusion based on health status

^a^Although individuals aged <16 years may be eligible, only those aged ≥16 years were included in this analysis due to the lack of comparator populations.

This study aimed to assess seroprevalence using 3 sources collected in parallel from November to December 2023 to evaluate consistency or potential differences across sampling methods.

## OUR STUDY

We conducted 3 SARS-CoV-2 seroprevalence surveys in Japan using residual serum samples from blood donors, commercial laboratories, and health checkups. The blood donor survey included 18 048 samples from individuals aged 16–69 years, collected across 47 prefectures between November 14 and 28, 2023. A commercial laboratory survey analyzed 3947 samples from individuals of all ages (≥0 years) across 22 prefectures, collected between November 25 and December 13, 2023. Of the 3947 specimens collected in the commercial laboratory survey, 3016 (76.4%) were from individuals aged 16 years or older. The health checkup survey examined 4258 samples from individuals aged ≥16 years across 30 prefectures, collected between November 28 and December 14, 2023. This study was conducted under the Act on the Prevention of Infectious Diseases and Medical Care for Patients with Infectious Diseases (Act No. 114 of 1998, Japan); therefore, no formal ethics review or participant consent was required.

First, the prefectures were matched; the main analysis included 15 prefectures where all 3 specimen sources were available. Then, the data were stratified by sex and age group (16–19, 20–29, 30–39, 40–49, 50–59, 60–69, and ≥70 years). To adjust for age and sex distribution by prefecture, we used a weighted tabulation approach [20] based on the baseline Japanese population data from October 2022. N antibodies were measured using the Elecsys Anti-SARS-CoV-2 assay (Roche, Basel, Switzerland), with a cutoff index of 1.0 U/mL. Overall seroprevalence was calculated for individuals aged 16–69 years within the 15 prefectures. We calculated 95% confidence intervals using the binomial exact method, with statistical significance set at *P* < .05. Within each age group, the differences in seroprevalence across the 3 surveys were assessed with a Fisher exact test, and the resulting *P* values were adjusted using the Benjamini-Hochberg false discovery rate (FDR) procedure. For strata showing FDR-adjusted significance, pairwise Fisher exact tests were additionally conducted with correction for multiple tests using Holm's method. Finally, we conducted a sensitivity analysis using 2 alternative sets of prefectures: (1) all available prefectures for each specimen source (unmatched) and (2) only prefectures that were matched to the health checkup sample (n = 30) to assess the robustness of the results.

**Figure 1.**
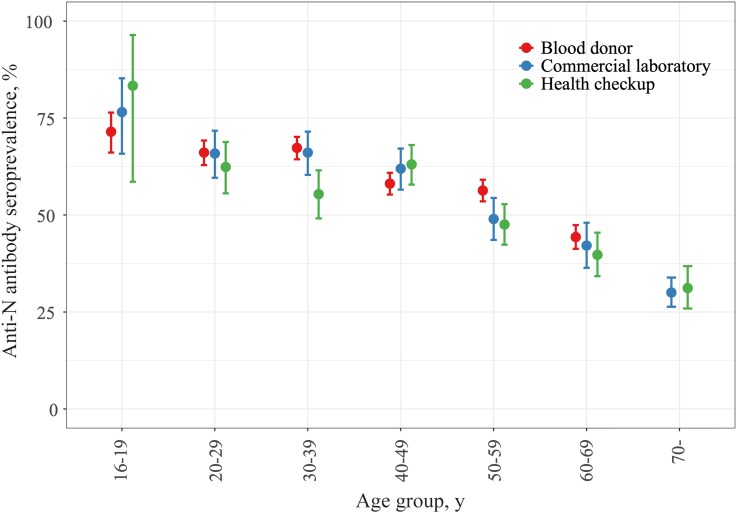
Comparison of infection-induced severe acute respiratory syndrome coronavirus 2 seroprevalence among blood donors, commercial laboratories, and health checkups. The analysis was limited to 15 prefectures with specimens available for all 3 sample populations. Seroprevalence estimates were adjusted for age and sex distribution in the population of each prefecture.

The seroprevalence among blood donors, commercial laboratories, and health checkup samples was 58.8% (57.6%–60.1%), 57.6% (55.2%–60.1%), and 53.5% (51.0%–56.0%), respectively (Figure 1; Supplementary Table 1). Younger age groups exhibited high seroprevalence, whereas older groups showed low and declining levels, consistent with our previous findings in Japan [6, 12]. Notable deviations were observed between 30–39 (lower seroprevalence among health checkup samples; *P* < .05) and 50–59 years (higher seroprevalence among blood donors; *P* < .05) age groups. Seroprevalence was higher among blood donors than health checkup samples in both the 30–39 and 50–59 age groups. Commercial lab samples resembled blood donors in the 30–39 age group and health checkups in the 50–59 age group. This pattern held in sensitivity analyses for the 30–39 age group but was less consistent for the 50–59 age group (Supplementary Figure 1).

This overall trend likely reflects the higher infection rates among younger populations and greater public health adherence among older populations [6]. The deviations in the 30–39 and 50–59 age groups, likely due to variations in infection risk within the sampled population, could not have been discovered in a single-source study. This emphasizes the importance of using multiple residual sample sources in seroepidemiological surveys. Moreover, this approach prompted further investigation into the factors contributing to these deviations [21].

This study has some limitations. First, the estimated seroprevalence proportion did not account for the sensitivity or waning of anti-N antibodies. However, this effect is minimal as the Roche assay used in this study demonstrates high sensitivity and specificity, with durability to detect past infections for up to 2 years in most individuals [22–24]. Moreover, N antibody seroprevalence in Japan was only 2.1% as of December 2021 [4], indicating few infections before 2022. This suggests that most infections during and after Omicron waves would be captured during our study period. Second, comparison of the 3 parallel surveys did not ensure their generalizability to a broader population. Finally, the analysis represented a single point in time, and the results may vary with SARS-CoV-2 infection dynamics.

In conclusion, multisource parallel surveys encompassing 26 253 blood samples offer a comprehensive view of SARS-CoV-2 seroprevalence. These findings offer crucial insights for public health decision-making and response strategies, providing valuable guidance for managing pandemics and shaping future policies.

## Supplementary Material

ofaf415_Supplementary_Data

## Supplementary Data

Supplementary materials are available at *Open Forum Infectious Diseases*  online. Consisting of data provided by the authors to benefit the reader, the posted materials are not copyedited and are the sole responsibility of the authors, so questions or comments should be addressed to the corresponding author.

## References

[1] Byambasuren O, Dobler CC, Bell K, et al Comparison of seroprevalence of SARS-CoV-2 infections with cumulative and imputed COVID-19 cases: systematic review. PLoS One 2021; 16:e0248946. 33798211 10.1371/journal.pone.0248946 PMC8018669

[2] Erikstrup C, Laksafoss AD, Gladov J, et al Seroprevalence and infection fatality rate of the SARS-CoV-2 omicron variant in Denmark: a nationwide serosurveillance study. Lancet Reg Health Eur 2022; 21:100479. 35959415 10.1016/j.lanepe.2022.100479 PMC9355516

[3] Keith RJ, Holm RH, Amraotkar AR, et al Stratified simple random sampling versus volunteer community-wide sampling for estimates of COVID-19 prevalence. Am J Public Health 2023; 113:768–77. 37200600 10.2105/AJPH.2023.307303 PMC10262242

[4] Arashiro T, Arai S, Kinoshita R, et al National seroepidemiological study of COVID-19 after the initial rollout of vaccines: before and at the peak of the Omicron-dominant period in Japan. Influenza Other Respir Viruses 2023; 17:e13094. 36824391 10.1111/irv.13094 PMC9890143

[5] Brynildsrud O . COVID-19 prevalence estimation by random sampling in population—optimal sample pooling under varying assumptions about true prevalence. BMC Med Res Methodol 2020; 20:196. 32703158 10.1186/s12874-020-01081-0 PMC7376319

[6] Kinoshita R, Arashiro T, Kitamura N, et al Infection-induced SARS-CoV-2 seroprevalence among blood donors, Japan, 2022. Emerg Infect Dis 2023; 29:1868–71. 37506681 10.3201/eid2909.230365 PMC10461656

[7] Chang L, Hou W, Zhao L, et al The prevalence of antibodies to SARS-CoV-2 among blood donors in China. Nat Commun 2021; 12:1383. 33654063 10.1038/s41467-021-21503-x PMC7925561

[8] Fischer B, Knabbe C, Vollmer T. SARS-CoV-2 IgG seroprevalence in blood donors located in three different federal states, Germany, March to June 2020. Euro Surveill 2020; 25:2001285. 32700672 10.2807/1560-7917.ES.2020.25.28.2001285 PMC7376847

[9] Jones JM, Opsomer JD, Stone M, et al Updated US infection- and vaccine-induced SARS-CoV-2 seroprevalence estimates based on blood donations, July 2020-December 2021. JAMA 2022; 328:298–301. 35696249 10.1001/jama.2022.9745 PMC9194752

[10] Slot E, Hogema BM, Reusken CB, et al Low SARS-CoV-2 seroprevalence in blood donors in the early COVID-19 epidemic in The Netherlands. Nat Commun 2020; 11:5744. 33184284 10.1038/s41467-020-19481-7 PMC7665189

[11] Uyoga S, Adetifa IM, Karanja HK, et al Seroprevalence of anti–SARS-CoV-2 IgG antibodies in Kenyan blood donors. Science 2021; 371:79–82. 33177105 10.1126/science.abe1916 PMC7877494

[12] Kinoshita R, Miyamoto S, Sakuraba S, et al Infection- and vaccine-induced SARS-CoV-2 seroprevalence, Japan, 2023. Emerg Infect Dis 2024; 30:1267–70. 38782366 10.3201/eid3006.231454 PMC11138984

[13] Clarke KE . Seroprevalence of infection-induced SARS-CoV-2 antibodies—United States, September 2021–February 2022. MMWR Morb Mortal Wkly Rep 2022; 71:606–8. 35482574 10.15585/mmwr.mm7117e3 PMC9098232

[14] Kao S-YZ, Nycz E, Benoit TJ, Clarke KE, Jones JM. Comparison of SARS-CoV-2 seroprevalence estimates between commercial lab serum specimens and blood donor specimens, United States, September–December 2021. Microbiol Spectr 2024; 12:e00123–4. 38869287 10.1128/spectrum.00123-24 PMC11302068

[15] Li M-C, Lee N-Y, Tsai W-L, Ko W-C. A seroprevalence study of COVID-19 at a campus in Southern Taiwan. J Microbiol Immunol Infect 2021; 54:1008–10. 33867282 10.1016/j.jmii.2021.03.018 PMC8028599

[16] Jacquot C, Tiberghien P, van den Hurk K, et al Blood donor eligibility criteria for medical conditions: a BEST collaborative study. Vox Sang 2022; 117:929–36. 35405021 10.1111/vox.13281

[17] Ministry of Health Labour and Welfare. 平成 24 年 労働者健康状況調査 [2012 Worker Health Status Survey]. **2013**. Available at: https://www.mhlw.go.jp/toukei/list/dl/h24-46-50_01.pdf. Accessed 16 December 2024.

[18] Kao SY, Nycz E, Benoit TJ, Clarke KEN, Jones JM. Comparison of SARS-CoV-2 seroprevalence estimates between commercial lab serum specimens and blood donor specimens, United States, September-December 2021. Microbiol Spectr 2024; 12:e0012324. 38869287 10.1128/spectrum.00123-24 PMC11302068

[19] Bajema KL, Dahlgren FS, Lim TW, et al Comparison of estimated severe acute respiratory syndrome coronavirus 2 seroprevalence through commercial laboratory residual sera testing and a community survey. Clin Infect Dis 2021; 73:e3120–3. 33300579 10.1093/cid/ciaa1804 PMC7799302

[20] Gideon L . Handbook of Survey Methodology for the Social Sciences. Springer; 2012.

[21] Kinoshita R, Miyamoto S, Suzuki T, Suzuki M, Yoneoka D. Interpreting the influence of using blood donor residual samples for SARS-CoV-2 seroprevalence studies in Japan: cross-sectional survey study. JMIR Public Health Surveill 2025; 11:e60467. 39931010 10.2196/60467 PMC11833190

[22] Navaratnam AMD, Shrotri M, Nguyen V, et al Nucleocapsid and spike antibody responses following virologically confirmed SARS-CoV-2 infection: an observational analysis in the virus watch community cohort. Int J Infect Dis 2022; 123:104–11. 35987470 10.1016/j.ijid.2022.07.053 PMC9385348

[23] Swartz MD, DeSantis SM, Yaseen A, et al Antibody duration after infection from SARS-CoV-2 in the Texas coronavirus antibody response survey. J Infect Dis 2022; 227:193–201. 10.1093/infdis/jiac167 PMC9833436 35514141

[24] Loesche M, Karlson EW, Talabi O, et al Longitudinal SARS-CoV-2 nucleocapsid antibody kinetics, seroreversion, and implications for seroepidemiologic studies. Emerg Infect Dis 2022; 28:1859–62. 35868337 10.3201/eid2809.220729 PMC9423917

