# Supplementary material for: Comparison of Infection-Induced SARS-CoV-2 Seroprevalence Across Large-Scale Residual Samples From Blood Donors, Commercial Laboratories, and Health Checkups in Japan, 2023
Source: Open Forum Infect Dis. 2025 Jul 17;12(8):ofaf415. doi: 10.1093/ofid/ofaf415 (PMC12321519; doi:10.1093/ofid/ofaf415)
Supplement: ofaf415_Supplementary_Data [file ofaf415_supplementary_data.docx]

**Supplementary Figure 1:** (A) The number of prefectures used is unmatched, with 30 prefectures for health checkups, 47 prefectures for blood donors, and 22 prefectures for commercial laboratories. (B) The number of prefectures used is matched to the health checkup sample, with 30 prefectures for both health checkup and blood donor samples.

**Supplementary Table 1:** Values of estimated seroprevalence shown in Figure 1. Prefectures included in Figure 1 were matched, with a total of 15 prefectures. Seroprevalence estimates were adjusted for age and sex distribution in the population of each prefecture.

|  | ****Blood donor**** |  | ****Commercial laboratory**** |  | ****Health checkup**** |  |
| --- | --- | --- | --- | --- | --- | --- |
| Age group (years) | % Positive (95% CI) | **n** | % Positive (95% CI) | **n** | % Positive (95% CI) | **n** |
| **Overall (16–69)** | **58.8% (57.6–60.1)** | 5760 | **57.6% (55.2–60.1)** | 1824 | **53.5% (51.0–56.0)** | 2300 |
| 16–19 | 71.5% (66.1–76.4) | 332 | 76.5% (65.8–85.2) | 248 | 83.3% (58.6–96.4) | 8 |
| 20–29 | 66.1% (62.9–69.2) | 583 | 65.9% (59.6–71.7) | 319 | 62.4% (55.6–68.8) | 164 |
| 30–39 | 67.3% (64.4–70.1) | 802 | 66.1% (60.4–71.5) | 322 | 55.4% (49.1–61.5) | 218 |
| 40–49 | 58.1% (55.3–60.9) | 1374 | 61.9% (56.5–67.1) | 311 | 63.1% (57.8–68.1) | 482 |
| 50–59 | 56.3% (53.5–59.1) | 1789 | 49.0% (43.6–54.4) | 322 | 47.6% (42.4–52.8) | 811 |
| 60–69 | 44.3% (41.3–47.4) | 880 | 42.1% (36.4–48.0) | 302 | 39.7% (34.2–45.5) | 617 |
| 70+ | NA | NA | 30.0% (26.4–33.9) | 618 | 31.2% (25.9–36.8) | 15 |
